# Evidence for Inhibitory Perturbations on the Amplitude, Gating, and Hysteresis of A-Type Potassium Current, Produced by Lacosamide, a Functionalized Amino Acid with Anticonvulsant Properties

**DOI:** 10.3390/ijms23031171

**Published:** 2022-01-21

**Authors:** Hsin-Yen Cho, Tzu-Hsien Chuang, Sheng-Nan Wu

**Affiliations:** 1 Department of Physiology, College of Medicine, National Cheng Kung University, Tainan City 70101, Taiwan; lilyzhou861126@gmail.com (H.-Y.C.); fytg55qq@gmail.com (T.-H.C.); 2 Institute of Basic Medical Sciences, College of Medicine, National Cheng Kung University, Tainan City 70101, Taiwan

**Keywords:** lacosamide (Vimpat^®^), A-type K^+^ current, current kinetics, voltage hysteresis, pituitary cell, hippocampal neuron

## Abstract

Lacosamide (Vimpat^®^, LCS) is widely known as a functionalized amino acid with promising anti-convulsant properties; however, adverse events during its use have gradually appeared. Despite its inhibitory effect on voltage-gated Na^+^ current (*I*_Na_), the modifications on varying types of ionic currents caused by this drug remain largely unexplored. In pituitary tumor (GH_3_) cells, we found that the presence of LCS concentration-dependently decreased the amplitude of A-type K^+^ current (*I*_K(A)_) elicited in response to membrane depolarization. The *I*_K(A)_ amplitude in these cells was sensitive to attenuation by the application of 4-aminopyridine, 4-aminopyridine-3-methanol, or capsaicin but not by that of tetraethylammonium chloride. The effective IC_50_ value required for its reduction in peak or sustained *I*_K(A)_ was calculated to be 102 or 42 µM, respectively, while the value of the dissociation constant (*K*_D_) estimated from the slow component in *I*_K(A)_ inactivation at varying LCS concentrations was 52 µM. By use of two-step voltage protocol, the presence of this drug resulted in a rightward shift in the steady-state inactivation curve of *I*_K(A)_ as well as in a slowing in the recovery time course of the current block; however, no change in the gating charge of the inactivation curve was detected in its presence. Moreover, the LCS addition led to an attenuation in the degree of voltage-dependent hysteresis for *I*_K(A)_ elicitation by long-duration triangular ramp voltage commands. Likewise, the *I*_K(A)_ identified in mouse mHippoE-14 neurons was also sensitive to block by LCS, coincident with an elevation in the current inactivation rate. Collectively, apart from its canonical action on *I*_Na_ inhibition, LCS was effective at altering the amplitude, gating, and hysteresis of *I*_K(A)_ in excitable cells. The modulatory actions on *I*_K(A)_, caused by LCS, could interfere with the functional activities of electrically excitable cells (e.g., pituitary tumor cells or hippocampal neurons).

## 1. Introduction

Lacosamide (LCS, Vimpat^®^) is regarded as a functionalized amino acid (available orally and intravenously) for the treatment of a wide variety of seizures, such as focal-onset seizure or refractory status epilepticus [1,2,3,4,5,6,7,8,9,10,11,12,13,14,15,16,17]. However, of note, although LCS is safe and effective in anti-convulsant activities, the unwanted events following LCS treatment, such as dizziness, abnormal vision, diplopia, ataxia, personality changes, and cardiovascular adverse events (e.g., sinus node dysfunction), have gradually emerged [3,6,7,18,19,20,21,22,23,24]. The risk of myoclonic seizure was also reported to occur during the treatment with this drug [25]. On the other hand, a previous report by Hagenacker et al. (2013) has shown the ability of LCS to reduce analgesic effects and limit the effect on *I*_Na_ inhibition in dorsal root ganglion neurons in a model of peripheral neuropathic pain [26]. Therefore, it is worthwhile to reappraise the ionic mechanism of LCS actions in varying types of transmembrane ionic currents, although the voltage-gated Na^+^ (Na_V_) channels were, until recently, focused on its therapeutic effectiveness [27,28,29,30,31,32].

Voltage-gated K^+^ (K_V_) channels are recognized to have essential roles in determining membrane excitability. K_V_ channels of the K_V_1.4 (KCNA4), K_V_3.3 (KCNC3), K_V_3.4 (KCNC4), and K_V_4.1-4.3 (KCND1-3) types have been regarded to be the main determinants of A-type K^+^ currents (*I*_K(A)_) (i.e., fast-inactivating K^+^ current or transient outward K^+^ currents [*I*_TO_]) [33,34]. The *I*_K(A)_ is viewed to be voltage-gated K^+^ currents that are sensitive to block by 4-aminopyridine (4-AP) and that undergo fast activation and inactivation in response to step depolarization [33,35,36,37]. It is known as an important regulator of cell excitability and firing pattern, which have been characterized in varying types of neurons, heart cells, and endocrine cells [33,34,35,36,38,39,40,41,42,43,44,45,46,47,48,49,50,51,52]. However, whether or how LCS and other related compounds cause any adjustments on the magnitude, gating kinetics, and voltage-dependent hysteresis (V_hys_) of *I*_K(A)_ has not been thoroughly elaborated, although its seeming ineffectiveness in altering the magnitude of K^+^ currents has been previously reported [30,53].

Therefore, in light of the above-mentioned considerations, the objective of the current study is to address the question of whether LCS can cause any possible modifications on other types of membrane ionic currents, such as *I*_K(A)_ in pituitary tumor (GH_3_) cells and mHippoE-14 hippocampal neurons, and to determine the gating kinetics of *I*_K(A)_ in the presence of LCS and to evaluate whether LCS interferes with the strength of voltage-dependent hysteresis (V_hys_) of *I*_K(A)_ activated by isosceles-triangular ramp pulse. GH_3_ cells are a clonal pituitary cell line that secretes both prolactin and growth hormone and have served as a useful model for investigations into the regulation of hormonal secretion [46,52], while the mHippoE-14 hippocampal cell line is known to possess the characteristics of embryonic hippocampal neurons and to enable accurate in-vitro assays for use in the discovery, development, and validation of new therapeutics targeted to central nervous system disorders [54]. Of importance, in this study, we provide substantial evidence to disclose that the exposure to LCS is capable of interacting with K_A_ channels to perturb the modifications on the magnitude, gating kinetics, and V_hys_ strength of *I*_K(A)_ present in electrically excitable cells (e.g., GH_3_ and mHippoE-14 cells). The inhibition by LCS of *I*_Na_ and *I*_K(A)_ may synergistically act to perturb the functional activities of excitable cells in culture or occurring in vivo.

## 2. Results

### 2.1. Inhibitory Effect of Lacosamide (LCS) on A-type K^+^ Current (I_K(A)_) Measured from Pituitary Tumor (GH_3_) Cells

**Figure 1:**
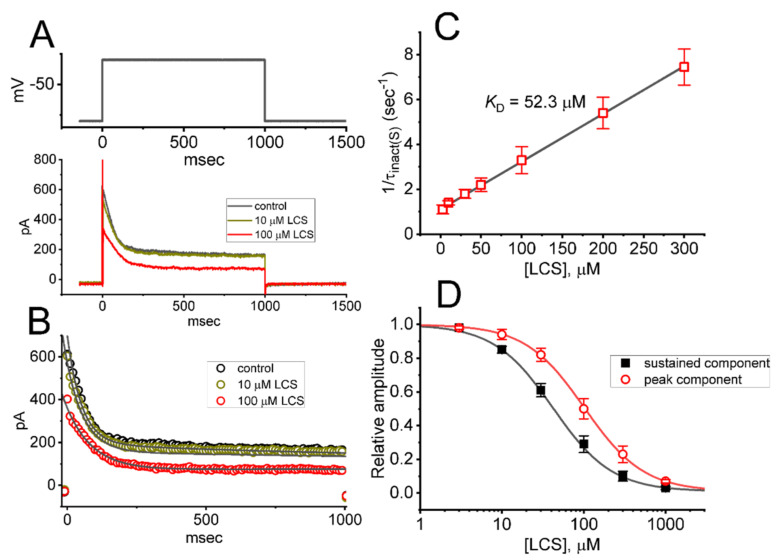
Effects of LCS on the magnitude of *I*_K(A)_ in GH_3_ pituitary tumor cells. These experiments were made in cells that were bathed in Ca^2+^−free, Tyrode’s solution containing 1 μM tetrodotoxin (TTX) and 0.5 mM CdCl_2_, and we backfilled the recording electrode by using a K^+^−containing (145 mM) solution. (**A**) Representative *I*_K(A)_ traces obtained in the control (black color) and during cell exposure to 10 μM LCS (brown color) or 100 μM LCS (red color). The uppermost part denotes the voltage−clamp protocol used. (**B**) Current traces showing an expanded record from those in (**A**); their trajectories were fitted by a two exponential (smooth gray line). Data points (indicated in open circles) were taken with or without the addition of LCS (10 or 100 μM). (**C**) Kinetic estimate in LCS−mediated block of *I*_K(A)_ in GH_3_ cells (mean ± SEM; *n* = 8 for each point). The reciprocal of the slow component in the inactivation time constant (1/*τ_inact(S)_*) of *I*_K(A)_ derived from the exponential fit of the I_K(A)_ trajectory was collated and then linearly plotted against the LCS concentration (gray straight line). Forward (on, *k*_+1_*) or backward (off, *k*_−1_) rate constant for the binding scheme, derived from the slope and the ordinate axis of the interpolated line, was 0.0213 s^−1^μM^−1^ or 1.112 s^−1^, respectively; thereafter, the *K*_D_ value (*k*_−1_/*k*_+1_* = 52.3 μM) was yielded. (**D**) Concentration-dependent relationship of LCS effect on peak (red open circles) or sustained (black filled squares) *I*_K(A)_ activated by 1−sec membrane depolarization (mean ± SEM; *n* = 8 for each point). Current amplitude was measured at the beginning or end-pulse of each depolarizing step, from −80 to −30 mV, with a duration of 1 s. The sigmoidal curve (black or red line) represents the best fit to the Hill equation (detailed in Materials and Methods). The statistical analyses were conducted with ANOVA−2 for repeated measures, (*p* (factor 1, groups among data taken at different LCS concentrations) < 0.05, *p* (factor 2, groups between the sustained and peak *I*_K(A)_) < 0.05, *p* (interaction) < 0.05, followed by post-hoc Fisher’s least−significance difference test, *p* < 0.05).

For the first stage of whole-cell current recordings, we intentionally kept GH_3_ cells in Ca^2+^-free, Tyrode’s solution containing 1 μM tetrodotoxin (TTX) and 0.5 mM CdCl_2_. The measurements were conducted in the recording electrode filled with a K^+^-containing (145 mM) solution. In order to elicit I_K(A)_, we voltage-clamped the examined cell at a holding potential of −80 mV, and a depolarizing pulse to −30 mV with a duration of 1 sec was thereafter applied to it. The main reason why the test potential was set at −30 mV is that as the test pulse was set beyond +10 mV, different types of high-threshold delayed-rectifier K^+^ currents with a slowly inactivating property could be evoked. Consequently, the effect on K^+^ currents exerted by LCS would have been contaminated. In keeping with previous observations in different cell types [35,36,37,50], the *I*_K(A),_ which biophysically displayed a rapidly activating and inactivating property, was consistently seen in GH_3_ cells. Upon membrane depolarization, the current residing in these cells was noticed to activate or inactivate in a monophasic or biphasic manner, respectively. Of interest, within one minute of exposing cells to LCS, the peak and sustained amplitudes of *I*_K(A)_ in response to long sustained depolarization were progressively decreased (Figure 1A). The time course of LCS-mediated inhibition of *I*_K(A)_ is illustrated in the Supplementary Materials file (Figure S1). For example, as cells were exposed to LCS at a concentration of 100 μM, the peak and sustained components of *I*_K(A)_ were noticeably reduced to 306 ± 23 and 85 ± 11 pA (*n* = 8, *p* < 0.05) from control values of 592 ± 32 and 191 ± 18 pA (*n* = 8), respectively. When LCS was removed, peak amplitude returned to 545 ± 21 pA (*n* = 8). As demonstrated in Figure 1A,B, the inactivation time course of *I*_K(A)_ in response to long step depolarization was evidently hastened. For example, during cell exposure to 100 μM LCS, the slow component of inactivation time constant (*τ_inact_*_(S)_) in *I*_K(A)_ was profoundly declined to 297 ± 35 ms (*n* = 8, *p* < 0.05) from a control value of 1004 ± 1021 ms (*n* = 8); however, no obvious change in the fast component of inactivation time constant of the current was demonstrated in its presence (56 ± 9 ms (in control) versus 53 ± 11 ms (in the presence of 100 μM LCS); *n* = 8, *p* > 0.05). The results reflect that the presence of LCS is able to exert a depressant action on *I*_K(A)_ in these cells.

### 2.2. Kinetic Estimate of LCS-Induced Block on I_K(A)_ in GH_3_ Cells

During cell exposure to LCS, in addition to the measured reduction in the amplitude of peak and sustained *I*_K(A)_, the slow component of the current inactivation rate during long depolarizing command voltage tended to become faster. Therefore, we set out to evaluate the inactivation time course of LCS-mediated block in situations where cells were exposed to different LCS concentrations (3–300 μM). The concentration-dependence of the slow component in the *I*_K(A)_ inactivation rate that we have obtained by adding different concentrations of this compound was then constructed and is illustrated in Figure 1B. The plot demonstrates data points tightly clustered around a straight line that rises up diagonally from the horizontal axis. In other words, its blocking effect on *I*_K(A)_ led to a concentration-dependent increase in the slow component of the current inactivation rate (i.e., 1/*τ_inact(S)_*). As a result, the LCS-perturbed effect on *I*_K(A)_ residing in GH_3_ cells can be reliably explained by a state-dependent blocking mechanism through which the LCS molecule can preferentially bind to and block the open state (conformation) of the A-type K^+^ (K_A_) channels. The first-order blocking scheme is shown in the following:

(1) $$C\begin{array}{c} \alpha \\ \;⇄\\ \beta \end{array}\;O\;\begin{array}{c} k_{+1}{}^{*}\cdot \left[LCS\right]\\ ⇄\\ k_{-1} \end{array}\;O\cdot \left[LCS\right]$$

Alternatively, the dynamical system, yielding three equations, becomes:

dCdt=−α×C+β×O

dOdt=α×C+k−1×O·LCS−O×β+k+1*·LCS

dO·LCSdt=−k−1×O·LCS+k+1*·LCS×O

where *α* or *β* is the kinetic constant for the opening or closing of the K_A_ channels underlying the *I*_K(A)_, respectively; *k*_+1_* or *k*_−1_ is the blocking (i.e., depending on the LCS concentration) or unblocking rate constant of the LCS binding, respectively; and [LCS] is the LCS concentration applied. C, O, or O·[LCS] indicates the closed (or resting), open, or open-bound state of the channel, respectively.

The forward (on, *k*_+1_*) or backward (off, *k*_−1_) rate constant was reliably estimated on the basis of the slow component in the inactivation time constant (*τ_inact(S)_*) of *I*_K(A)_ during cell exposure to different LCS concentrations, as described under the Materials and Methods (Figure 1C). Consequently, the forward or reverse rate constant was given to be 0.0213 μM^−1^s^−1^ or 1.112 s^−1^, respectively; thereafter, dividing *k*_−1_ by *k*_+1_* gave a dissociation constant (*K*_D_) of 52.3 μM.

As demonstrated in Figure 1D, the concentration-dependent relationship of LCS on peak and sustained *I*_K(A)_ evoked by 1 s long sustained depolarization was plotted. According to a least-squares fit to the modified Hill equation, the effective IC_50_ value required for LCS-mediated inhibitory effect on peak or sustained *I*_K(A)_ was estimated to be 102 or 42 μM, respectively, with a Hill coefficient of 1.2. Of note, the value needed to suppress the sustained *I*_K(A)_ amplitude in GH_3_ cells was nearly close to the K_D_ value computed on the basis of the first-order reaction scheme (Figure 1C), although the IC_50_ value for its inhibition of peak *I*_K(A)_ was higher than the *K*_D_ value (i.e., a two-fold increase).

### 2.3. Effect of LCS, Tetraethylammonium Chloride (TEA), 4-Aminopyridine-3-methanol (4-AP-3-MeOH), 4-Aminopyridine (4-AP), Capsaicin (Cap) on the Amplitude of I_K(A)_ in GH_3_ Cells

**Figure 2:**
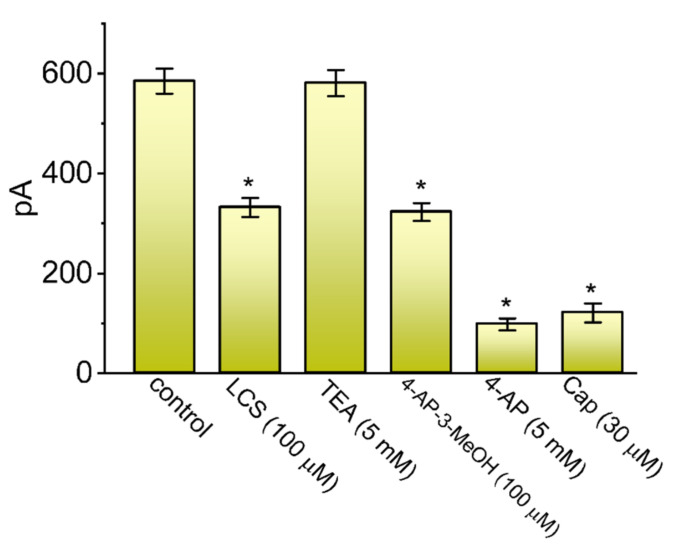
Effect of LCS, tetraethylammonium chloride (TEA), 4-aminopyridine-3-methanol (4-AP-3-MeOH), 4-aminopyridine (4-AP), or capsaicin (Cap) on the peak amplitude of *I*_K(A)_ identified in GH_3_ cells. In these experiments, we bathed GH_3_ cells in Ca^2+^-free, Tyrode’s solution, and the recording electrode was filled with K^+^-enriched (145 mM) solution. Current amplitude was measured at the start of the depolarizing pulse, from −80 to −30 mV. Each bar represents the mean ± SEM (*n* = 8). Data analysis was performed by *ANOVA-1* (*p* < 0.05). * Significantly different from control (*p* < 0.05).

We extended to test how the *I*_K(A)_ magnitude in these cells can be affected by different blockers of K_A_ channels. This set of experiments was conducted when the examined cells were depolarized from −80 to −30 mV with a duration of 1 s, and we thereafter measured current amplitude at the start of command voltage during cell exposure to different compounds. As shown in Figure 2, the presence of TEA (5 mM) failed to decrease the peak amplitude of *I*_K(A)_ in GH_3_ cells, while that of LCS (100 μM), 4-AP-3-MeOH (100 μM), 4-AP (5 mM), or Cap (30 μM) suppressed peak *I*_K(A)_ effectively. 4-AP-3-MeOH, a derivative of 4-AP, has been previously demonstrated to inhibit *I*_K(A)_ and to restore axonal conduction in injured spinal cord white matter [55,56], while Cap was reported to inhibit *I*_K(A)_ in heart cells [36,38]. Hence, similar to the effects of 4-AP, 4-AP-3-MeOH, or Cap, the ability of LCS to suppress the peak component of *I*_K(A)_ is clearly demonstrated in GH_3_ cells.

### 2.4. Effect of LCS on the Current versus Voltage (I–V) Relationship of I_K(A)_ Recorded from GH_3_ Cells

**Figure 3:**
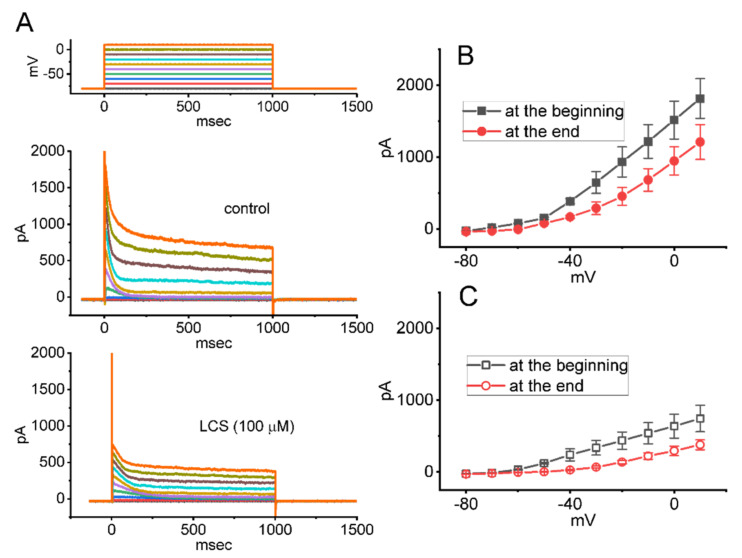
Effect of LCS on the current versus voltage (*I−V*) relationship of *I*_K(A)_ identified in GH_3_ cells. (**A**) Representative current traces obtained in the control period (i.e., LCS was not present, top panel) and during the exposure to 100 μM LCS (bottom panel). The uppermost part shows the voltage−clamp protocol applied. In (**B**,**C**), mean *I−V* relationships of peak or sustained *I*_K(A)_ achieved in the absence (filled symbols) and presence (open symbols) of 100 μM LCS are respectively illustrated (mean ± SEM; *n* = 7 for each point). The amplitude of peak or sustained *I*_K(A)_ was respectively measured at the beginning− or end−pulse of step depolarization from −80 to −30 mV with a duration of 1 s. The statistical analyses were made by *ANOVA−2* for repeated measures, (*p* (factor 1, groups among data taken at different level of membrane potentials) < 0.05, *p* (factor 2, groups between the absence and presence of LCS) < 0.05, *p* (interaction) < 0.05, followed by post−hoc Fisher’s least−significance difference test, *p* < 0.05). Of notice, no change in the overall *I−V* relationship of peak or sustained *I*_K(A)_ was detected in the presence of LCS, despite its reduction in the amplitude of peak or sustained *I*_K(A)_.

To characterize the inhibitory effect of LCS on peak and sustained *I*_K(A)_, we continued to explore whether this drug might exert any adjustments on the steady-state *I–V* relationship of peak *I*_K(A)_ inherently in these cells. The experiments were conducted in a voltage-clamp mode with a holding potential of −80 mV, and different rectangular voltages, ranging between −80 and +10 mV with a duration of 1 s, were subsequently applied to the examined cell. Figure 3A depicts representative *I*_K(A)_ traces taken with or without the application of LCS at a concentration of 100 μM, and the mean *I–V* relationships of peak or sustained *I*_K(A)_ in the absence and presence of this drug were established and are depicted in Figure 3B,C, respectively. The data indicated that no obvious adjustments in the overall *I–V* relationship of peak or sustained *I*_K(A)_ were detected during GH_3_-cell exposure to LCS, although the conductance of peak or sustained *I*_K(A)_ obtained in its presence was greatly reduced throughout the entire range of clamping potentials applied.

### 2.5. Steady-State Inactivation Curve of I_K(A)_ Obtained in the Absence and Presence of LCS

**Figure 4:**
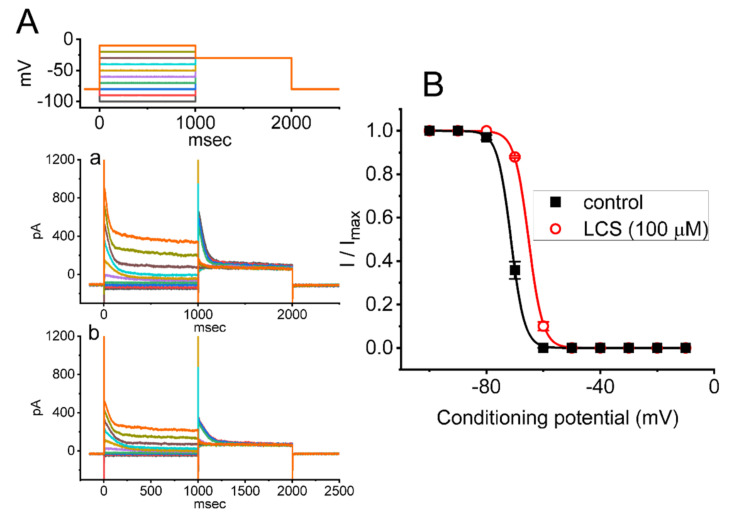
Effect of LCS on the quasi−steady−state inactivation curve of *I*_K(A)_ in GH_3_ cells. In this set of experiments, the conditioning voltage pulses with a duration of 1 s to a series of command voltage steps, ranging from −100 to −10 mV in 10−mV increments, were delivered to the examined cell from a holding potential of −80 mV. Following each conditioning pulse, a test pulse to −30 mV with a duration of 1 s was applied to evoke *I*_K(A)_. (**A**) Representative current traces obtained in the absence (a) and presence (b) of 100 μM LCS. The voltage−clamp protocol is illustrated in the uppermost part. (**B**) Steady-state inactivation curve of *I*_K(A)_ achieved in the absence (■) and presence (○) of 100 μM LCS (mean ± SEM; *n* = 7 for each point). The smooth curves were well fitted by the modified Boltzmann equation, as defined in Materials and Methods. The statistical analyses were made by *ANOVA−2* for repeated measures, (*p* (factor 1, groups among data taken at the conditioning voltage levels) < 0.05, *p* (factor 2, groups between the absence and presence of LCS) < 0.05, *p* (interaction) < 0.05, followed by post−hoc Fisher’s least−significance difference test, *p* < 0.05). Notice that the exposure to LCS shifts the midpoint of the inactivation curve along the voltage axis toward less hyperpolarized voltage (i.e., to the right); however, it is devoid of modifications in the gating charge of the curve in its presence.

To further characterize the inhibitory effect of LCS on *I*_K(A)_, we tested the voltage dependence of LCS effect on *I*_K(A)_ in GH_3_ cells by using a two-step voltage protocol. Figure 4A,B show the steady-state inactivation curve of *I*_K(A)_, taken with or without the application of 100 μM LCS. This set of current measurements was conducted with the voltage protocol in which a 1-s conditioning pulse to different potentials was applied to precede the 1-s test pulse to −30 mV from a holding potential of −80 mV. We then constructed the relationship between the conditioning potential and the normalized amplitude of peak *I*_K(A)_. The experimental data sets were collected and then least-squares fitted with a Boltzmann function (detailed in Materials and Methods). The values of either *V*_1/2_ or *q* (i.e., apparent gating charge) in the absence and presence of 100 μM LCS are either −71.4 ± 2.1 and −65.2 ± 1.9 mV or 10.2 ± 0.8 and 10.5 ± 0.9 *e* (*n* = 7), respectively. From these results, we were, therefore, able to show that the application of 100 μM LCS significantly shifted the midpoint of the inactivation curve toward the depolarizing voltage by approximately 6 mV (*p* < 0.05); conversely, no modifications in the gating charge of the curve were detected in the presence of this drug. Thus, in GH_3_ cells, there was a voltage dependence of the steady-state inactivation curve of *I*_K(A)_ during exposure to LCS.

### 2.6. Effect of LCS on the Recovery of I_K(A)_ Block Measured from GH_3_ Cells

**Figure 5:**
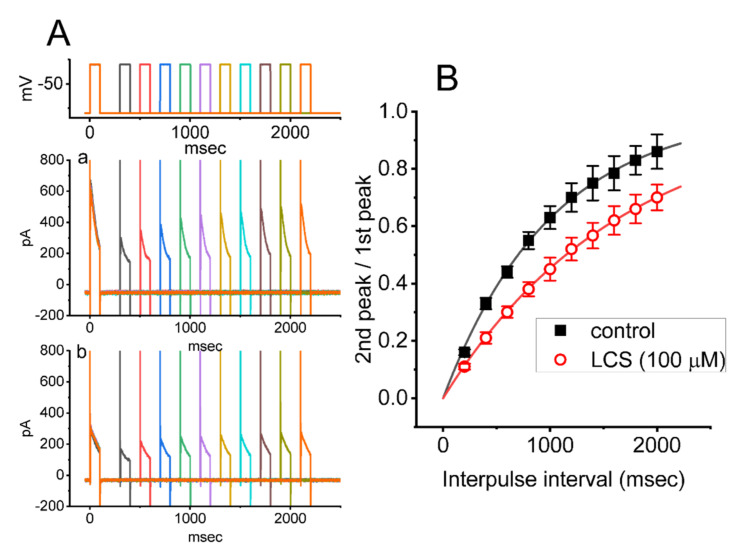
LCS−induced prolongation in the recovery of the *I*_K(A)_ block in GH_3_ cells. Cells were bathed in Ca^2+^−free, Tyrode’s solution, and we filled up the electrode with K^+^−containing solution. During the recordings, we applied another set of two-pulse voltage−clamp protocol to the examined cells. (**A**) Representative current traces, demonstrating current recovery from the block by use of two−step voltage protocol with varying inter−pulse intervals (as indicated in the uppermost part). a: control; b: in the presence of 100 μM LCS. The uppermost part shows the voltage−clamp protocol applied. (**B**) Time course of recovery from *I*_K(A)_ inactivation taken in the control period (■) and during exposure to 100 μM LCS (○) (mean ± SEM; *n* = 8 for each point). The smooth line, with or without the addition of LCS, was well fitted by single exponential function. The statistical analyses were made by *ANOVA−2* for repeated measures, (*p* (factor 1, groups among data taken at different inter-pulse interval) < 0.05, *p* (factor 2, groups between the absence and presence of LCS) < 0.05, *p* (interaction) < 0.05, followed by post−hoc Fisher’s least−significance difference test, *p* < 0.05).

In attempts to evaluate LCS-mediated inhibition of peak *I*_K(A)_ in these cells, we further investigated the recovery time course of current inactivation acquired with or without the application of this drug. For this set of current recordings, we implemented another two-pulse protocol that comprises a first (conditioning pulse) depolarizing voltage command and a second depolarizing command (test pulse) applied following varying inter-pulse intervals (Figure 5). The ratios of the peak *I*_K(A)_ in response to the second and first pulses were then taken as a measure of recovery from the *I*_K(A)_ block, and they were constructed and plotted versus inter-pulse intervals. The results, showing *I*_K(A)_ recovery from the block in the absence or presence of 100 μM LCS, are illustrated in Figure 5A,B. The time course, taken either from the control period (i.e., LCS was not present) or during exposure to 100 μM LCS, could be optimally described by a single exponential function with the recovery time constant of 1.01 ± 0.05 s (*n* = 8) or 1.66 ± 0.04 s (*n* = 8), respectively. It is, therefore, plausible to reflect, from the present observations, that the recovery of the *I*_K(A)_ block was overly prolonged by cell exposure to LCS. The delayed recovery of the *I*_K(A)_ block caused by LCS presence was most likely attributed to the open channel block.

### 2.7. Effect of LCS on Voltage-Dependent Hysteresis (V_hys_) of I_K(A)_ Identified in GH_3_

**Figure 6:**
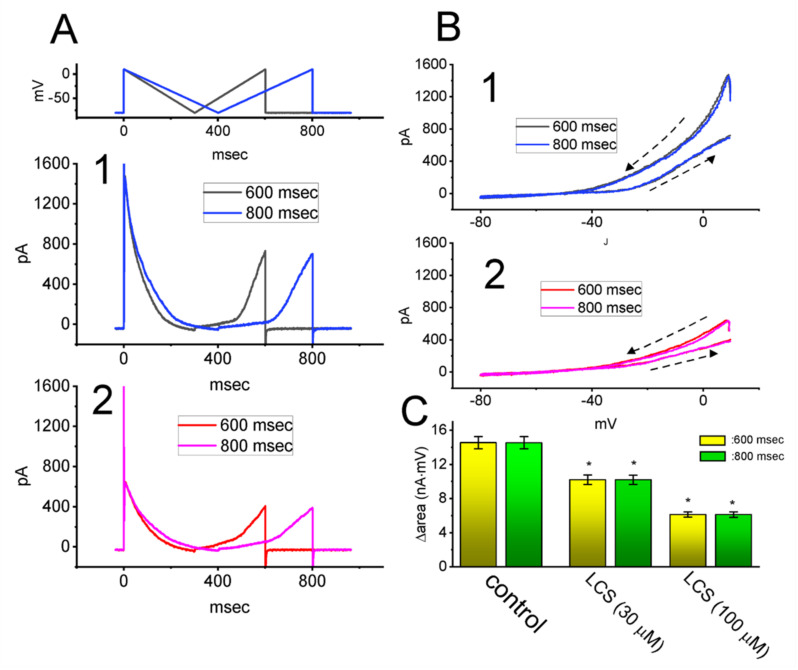
Inhibitory effect of LCS on the voltage-dependent hysteresis (V_hys_) of *I*_K(A)_ activated by inverted isosceles−triangular ramp pulse in GH_3_ cells. In this set of whole−cell current recordings, the potential applied to the examined cell was maintained at −80 mV, and we then imposed an inverted isosceles−triangular ramp pulse with the duration of 600 or 800 ms (i.e., ramp speed of ±150 or ±112.5 mV/s) to activate *I*_K(A)_ in response to descending (from +10 to −80 mV) and ascending (from −80 to +10 mV) ramp voltage−clamp commands. (**A**) Representative current traces obtained in the control period (**A1**) and during cell exposure to 100 μM LCS (**A2**). The uppermost part shows the voltage−clamp protocol applied. The black and blue colors in (**A1**) or red and pink colors in (**A2**) indicate the current traces obtained with the ramp duration of 600 and 800 ms, respectively. (**B**) Representative instantaneous *I−V* relationships of *I*_K(A)_ in response to inverted isosceles−triangular ramp pulse with a duration of 600 ms (black color in (**B1**) or red color in (**B2**)) or 800 ms (blue color in (**B1**) or pink color in (**B2**)). In (**B1**) and (**B2**), the instantaneous *I−V* relationships of *I*_K(A)_ are illustrated in the absence and presence of 100 μM LCS, respectively. The dashed arrow in (**B1**) and (**B2**) shows the direction of *I*_K(A)_ trajectories in which time passes during current elicitation by the inverted isosceles−triangular ramp pulse with a duration of 600 or 800 ms. (**C**) Summary bar graph demonstrating effects of LCS (30 or 100 μM) on the hysteretic area (Δ_area_) of *I*_K(A)_ activated by isosceles−triangular ramp pulse (mean ± SEM; *n* = 8 for each bar). The hysteretic area indicates the one under the curve activated during the descending and ascending ends of the triangular ramp pulse. Red or green bars respectively indicate the hysteretic areas taken in the duration of 600 or 800 ms. Data analysis was made by *ANOVA−1* (*p* < 0.05). Of note, there was an evident occurrence of voltage−dependent hysteresis (V_hys_) for *I*_K(A)_ activated by triangular ramp pulse, and the presence of LCS was able to attenuate the hysteretic area of the current. * Significantly different from controls (*p* < 0.05).

A number of studies have demonstrated the ability of *I*_K(A)_ strength to have impacts on varying firing patterns or action potential configurations existing in different types of electrically excitable cells [33,34,36,38,43,44,45,47,48,50,51,52,55]. For these reasons, we sought to examine the presence of *I*_K(A)_-linked V_hys_ and to explore whether cell exposure to LCS is capable of modifying the V_hys_ extent of *I*_K(A)_ activated in response to inverted isosceles-triangular ramp pulse with varying durations. In these experiments, in the absence and presence of LCS, we held the examined cell at −80 mV and a downsloping (forward) limb from +10 to −80 mV, followed by an upsloping (backward) limb back to +10 (i.e., inverted isosceles-triangular ramp pulse), with varying durations (600 or 800 ms) thereafter imposed on it (Figure 6A1,A2). Under these experimental conditions, the voltage-dependent hysteresis (V_hys_) of *I*_K(A)_ elicited by such a ramp waveform was robustly observed. Of note, the instantaneous *I*_K(A)_ amplitudes measured at the descending (ramping from +10 to −80 mV) limb of the triangular ramp were actually higher than those at the equipotential level of the ascending (ramping from −80 to +10 mV) limb of the ramp, especially at the voltages ranging between −40 and +10 mV (Figure 6B). The inactivation process of the current resulted in residual currents at the end of the ramp pulse, and the *I–V* loop in Figure 6B tended to be open when the voltage was ramped back to +10 mV.

Additional, during cell exposure to 30 or 100 μM LCS, the V_hys_ strength (i.e., Δarea (the difference in the area encircled by the curve in the descending and ascending direction)) of *I*_K(A)_ responding to both descending and ascending limbs of inverted triangular ramp pulse was progressively declined with increasing LCS concentration (Figure 6B,C). For example, as the duration of isosceles-triangular ramp applied was set at 800 ms (i.e., the ramp speed of ±112.5 mV/s), the value of Δarea for the V_hys_ emergence in the control period (i.e., LCS was not present) was 14.55 ± 0.72 nA·mV (*n* = 8), while in the same duration, the Δarea value was substantially declined to 6.13 ± 0.32 nA·mV during cell exposure to 100 μM LCS (*n* = 8, *p* < 0.05). Findings from these results, therefore, prompted us to indicate that there was apparently the emergence of V_hys_ for *I*_K(A)_ activation in response to an inverted isosceles-triangular ramp pulse in GH_3_ cells and that the hysteretic strength of the current became evidently diminished with increasing LCS concentration.

### 2.8. Inhibitory Effect of LCS on I_K(A)_ Present in mHippoE-14 Neurons

**Figure 7:**
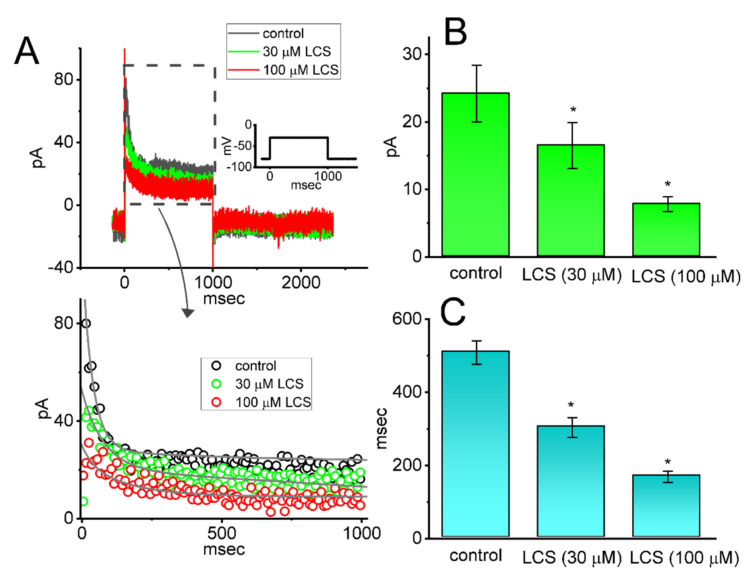
Effect of LCS on *I*_K(A)_ identified in mouse mHippoE−14 hippocampal neurons. Cells were bathed in Ca^2+^−free, Tyrode’s solution, and the recording electrode was filled with K^+^−enriched (145 mM) solution. (**A**) Representative current traces obtained in the absence (black color) and presence of 30 μM LCS (green color) or 100 μM LCS (red color). The inset denotes the voltage−clamp protocol given. The bottom panel shows an expanded record from the dashed box in the top panel. The data points (open circles) in each current trajectory were reduced by 50 for better illustration. The current trajectory was fitted with the goodness of fits by a two−exponential function (indicated by gray smooth line). In (**B**) or (**C**), the summary bar graph depicts the LCS effect on the sustained *I*_K(A)_ amplitude or *τ_inact(S)_* value of *I*_K(A)_ inactivation, respectively (mean ± SEM; *n* = 8). Current amplitude was activated by membrane depolarization from −80 to −30 mV with a duration of 1 s, and each current trajectory was fitted by a two−exponential function. Statistical analyses in (**B**,**C**) were made by *ANOVA−1* (*p* < 0.05). * Significantly different from controls (*p* < 0.05).

In a final set of whole-cell recordings, we decided to examine whether *I*_K(A)_ was functionally expressed in other types of cells (e.g., mHippoE-14 neurons) and how its magnitude or gating can be influenced by the presence of LCS. Similar to the experimental protocol done above in GH_3_ cells, cells were bathed in Ca^2+^-free, Tyrode’s solution containing 1 μM TTX and 0.5 mM CdCl_2_. As demonstrated in Figure 7, when the examined cell was 1-s depolarized from −80 to −30 mV, the *I*_K(A)_ with a rapid activating and inactivating property was robustly identified. This current was subject to inhibition by 5 mM 4-AP. Noticeably, when mHippoE-14 neurons were continually exposed to LCS (30 or 100 μM), the peak or sustained amplitude of *I*_K(A)_ in response to 1-s membrane depolarization from −80 to −30 mV was reduced. Concurrently, the LCS’s inhibition was accompanied by an evident reduction in the *τ_inact(S)_* value of *I*_K(A)_, with no change in the fast component of the inactivation time constant. For example, one minute after LCS (100 μM) was added, sustained *I*_K(A)_ amplitude was significantly reduced from 24.2 ± 4.2 to 7.8 ± 1.1 pA (*n* = 8, *p* < 0.05) and the *τ_inact(S)_* value was concurrently diminished from 509 ± 32 to 169 ± 15 ms (*n* = 8, *p* < 0.05). After washout of the drug, sustained *I*_K(A)_ was restored to 22.9 ± 3.9 pA (*n* = 7, *p* < 0.05). As such, consistent with the experimental observations described above in GH_3_ cells, we observed that the *I*_K(A)_ found in mHippoE-14 neurons was readily evoked in response to long sustained depolarization and that the current was sensitive to block by LCS.

## 3. Discussion

In the current study, we found that the presence of LCS could depress *I*_K(A)_ in a concentration-, time-, state-, and hysteresis-dependent manner in GH_3_ cells. The block of *I*_K(A)_ produced by LCS apparently is noted to be not inherently instantaneous, but it develops over time after the K_A_ channels become opened; thereafter, such a block produces a concurrent rise in the inactivation rate of the current activated by long-lasting membrane depolarization. During cell exposure to LCS, the *I*_K(A)_ exhibited a blunted peak and hastened decay, which is consistent with the possibility that the opening of channels was retarded by the binding of LCS. It is thus most likely that the blocking site of this drug is located within the channel pore only when the K_A_ channel is opened.

Distinguishable from previous observations [30,53], the present results demonstrated that LCS exerted a differential depressant action on the peak and sustained *I*_K(A)_ natively expressed in GH_3_ cells. *I*_K(A)_ in these cells is biophysically characterized by a rapidly activating and inactivating property during depolarizing voltage command, and it is sensitive to blockage by 4-AP, 4-AP-3-MeOH, or Cap but not by TEA (Figure 2). LCS tends to be selective for sustained over peak *I*_K(A)_ in response to a long depolarizing pulse. The rate of *I*_K(A)_ inactivation was considerably enhanced as the LCS concentration increased. Moreover, according to quantitative estimates from a heuristic reaction scheme elaborated above, the *K*_D_ (i.e., *k*_−1_/*k*_+1_*) value on the basis of the LCS-perturbed inactivation time constant of *I*_K(A)_ (i.e., *τ_inact(S)_*) seen in GH_3_ cells was yielded to be 52.3 μM. This value agrees closely with the calculated IC_50_ value (42 μM), which was needed for LCS-mediated inhibition of sustained *I*_K(A)_; however, it is apparently lower than the IC_50_ (102 μM) required for LCS’s ability to suppress peak *I*_K(A)_ (Figure 1). Therefore, the results strongly reflect that there is a considerable and selective block of sustained *I*_K(A)_ in the presence of LCS.

As reported previously, the effect of LCS on hNa_V_1.5 Na^+^ channels had an inhibitory effect, with a IC_50_ value of around 70–80 µM [29]. The estimated IC_50_ values required for inhibition by LCS of Na_V_1.7-, Na_V_1.3-, and Na_V_1.8-encoded currents were reported to be 182, 415, and 16 µM, respectively [57]. The IC_50_ value for LCS-mediated inhibition of *I*_K(A)_ was thus noticed to overlap those for inhibition of the different isoforms of Na_V_ channels. A previous report also showed that the mean LCS level in serum or CSF was around 8.2 µg/mL (33 µM) or 7.4 µg/mL (29 µM), respectively [58]. It is reasonable to assume, therefore, that the inhibitory effect on *I*_K(A)_ caused by LCS could be of pharmacological or therapeutic relevance.

Another point that needs to be explored is that the inactivation time course of the *I*_K(A)_ obtained in the presence of LCS was noticed to become faster in a concentration-dependent manner, although the initial rising phase of the current (i.e., activation time course) remained unaffected. That is, during exposure to LCS, the inactivation time course of *I*_K(A)_, especially in the slow component, had the propensity to decay more rapidly, although the fast component of current inactivation in response to long step depolarization failed to be changed. This feature can therefore be incorporated into a simple kinetic scheme (i.e., closed ↔ open ↔ open-bound), as stated above in the Results section. The results, in turn, mean that aside from its inhibition of peak *I*_K(A)_, the LCS molecule may primarily interact on the activation and/or inactivation process, presumably resulting in the adjustments of the magnitude and gating kinetics of the current, although the detailed underlying ionic mechanism of its action on *I*_K(A)_ remains to be delineated. However, the possibility that LCS may have a higher affinity for the open conformation of K_A_ channels than the closed (resting) conformation without evoking open channel block cannot be excluded.

The nonlinear V_hys_ phenomenon inherently in different types of voltage-gated ionic currents has been since demonstrated to play roles in affecting various electrical behavior of excitable cells [59,60]. In this study, we were able to identify the V_hys_ existence of *I*_K(A)_ residing in GH_3_ cells (Figure 6), implying that the voltage sensitivity inherent in the gating charge movement of K_A_ channels is thought to rely on the previous state of the channel [61,62]. In other words, the magnitude of *I*_K(A)_ is most likely to be contingent on the pre-existing state(s) or conformation(s) of the K_A_ channel. The V_hys_ strength of *I*_K(A)_ is important, and it would be engaged in the regulation of electrical behaviors of excitable cells such as GH_3_ cells [62,63]. In other words, as the action potential develops, the voltage dependence of K_A_ channels may shift the mode of V_hys_ to one in which activation occurs at more negative potentials, resulting in an increase in membrane repolarization, while as the membrane becomes negative, the voltage-dependence of *I*_K(A)_ activation would switch to more positive voltages, therefore enhancing cell excitability [44,61].

In this study, we additionally evaluated the possible perturbations by LCS of the instantaneous and non-equilibrium property inherent in *I*_K(A)_ activated by an inverted isosceles-triangular ramp pulse. The experimental observations that we have obtained led us to unravel the LCS’s effectiveness in reducing the V_hys_-linked Δarea for *I*_K(A)_ elicited by a triangular ramp pulse, suggesting that V_hys_ behavior is engaged in the voltage-dependent activation of the current. During the exposure to LCS, the steady-state inactivation curve of *I*_K(A)_ was shifted to less hyperpolarized potential (Figure 4), and the recovery of the current block also became slowed (Figure 5). In this scenario, it is reasonable to assume that any modifications by LCS of *I*_K(A)_ depend not only on the LCS concentration given but also on various confounding factors, such as the pre-existing level of the resting potential and the different firing patterns of action potentials or their combinations, assuming the magnitude of *I*_K(A)_ is sufficiently present in the cells examined.

From the present observations, we show that LCS shifted the inactivation curve of *I*_K(A)_ in the rightward direction, suggesting that it interacts with channels in the inactivated state. Consequently, neither the resting membrane potential nor the magnitude of *I*_Na_ could be seriously altered by its inhibitory effect on *I*_K(A)_, although it per se inhibited *I*_Na_ directly [27,28,29,30,31,32,57]. It has been established that the K_V_ channels, such as K_A_ channels, are crucial in shaping action potentials. Indeed, as its subthreshold activation and transient inactivation, the overall properties of *I*_K(A)_ make it an excellent target for any modulatory mechanism influencing membrane excitability and action potential firing. While the detailed mechanism of the LCS-induced suppression of *I*_K(A)_ remains unknown, our study suggests that the LCS-induced decrease in *I*_K(A)_ might increase excitability in electrically excitable cells, possibly leading to changes in neural plasticity [33,34,35,36,39,40,41,42,43,44,45,46,47,48,49,50,51,52].

The amplitude of *I*_K(A)_ observed in GH_3_ or mHippoE14 cells is sensitive to inhibition by 4-AP or 4-AP-3-MeOH, yet not by TEA. The K_A_-channel currents demonstrated here are thus primarily coded by genes from the K_V_4 (KCND) subfamily (e.g., K_V_4.2 and K_V_4.3) [35,42,46,64,65], although it is also likely that K_V_1.4, K_V_3.3, K_V_3.4, K_V_4.1, K_V_4.2, and K_V_4.3 contribute to the formation of K_A_ channels [46,52,65].

In this study, the *I*_K(A)_ residing in mHippoE-14 hippocampal neurons was also observed to be sensitive to blockage by LCS, and the inactivation time course of the current in response to long sustained depolarization was accompanied by a rapid decay. (Figure 7). The magnitude of *I*_K(A)_ has been previously demonstrated to be engaged in cell excitability, network synchronicity, and convulsant properties [33,34,43,45,47,48,49,51,55]. As a result, it is plausible to assume, from the present observations, that the LCS-mediated inhibition of *I*_K(A)_ would be of clinical, therapeutic, pharmacological, or even toxicological relevance, although a previous report showed that neither hERG nor L-type Ca^2+^ currents were altered by LCS [66]. The extent to which the unwanted reactions linked to the LCS treatment was explained by its perturbations on the magnitude, gating, and V_hys_ of *I*_K(A)_ and thus warrants further investigation. Findings from the present study tend to be informative as they highlight the proof-of-concept that needs to be taken into consideration since the wide spectrum of LCS’s beneficial effects has been clinically observed [1,2,3,4,5,6,7,9,10,11,12,13,14,15,16,17,18,19,20,21,24,25,32].

## 4. Materials and Methods

### 4.1. Chemicals, Drugs, and Solutions Used in This Study

Lacosamide (LCS, erlosamide, harkoseride, SPM 927, ADD 234034, Vimpat^®^, *R*-enantiomer of 2-acetamido-*N*-benzyl-3-methoxy-propioniamide, 2,3-diaminomaleonitrile, C_13_H_18_N_2_O_3_; https://pubchem.ncbi.nlm.nih.gov/compound/Lacosamide accessed on 10 January 2022), 4-aminopyridine (4-AP), capsaicin (Cap), tetraethylammonium chloride (TEA), and tetrodotoxin (TTX) were supplied by Sigma-Aldrich (Merck, Taipei, Taiwan), while 4-aminopyridine-3-methanol (4-AP-3-MeOH) was provided by Alfa Aesar (Uni-onward Corp., Tainan, Taiwan). For cell preparations, culture media, fetal bovine serum, L-glutamine, trypsin/EDTA, and penicillin–streptomycin were acquired from HyClone^TM^ (Thermo Fisher, Kaohsiung, Taiwan), whereas all other chemicals, such as aspartic acid, CdCl_2_, EGTA, and HEPES, were of laboratory grade and taken from standard sources.

The ion composition of the extracellular solution, (i.e., normal Tyrode’s solution buffered by HEPES) used for this study was as follows (in mM): NaCl 136.5, KCl 5.4, CaCl_2_ 1.8, MgCl_2_ 0.53, glucose 5.5, and HEPES-NaOH buffer 5 (pH 7.4). For the recordings of the flow through macroscopic *I*_K(A)_, we kept cells bathed in Ca^2+^-free, Tyrode’s solution in order to avoid the contamination of Ca^2+^-activated K^+^ and voltage-gated Ca^2+^ currents, while the patch electrodes for recording were backfilled with the following intracellular solution (in mM): K-aspartate 130, KCl 20, MgCl_2_ 1, KH_2_PO_4_ 1, Na_2_ATP_3_, Na_2_GTP 0.1, EGTA 0.1, and HEPES-KOH buffer 5 (pH 7.2). To record voltage-gated Na^+^ current (*I*_Na_), we substituted K^+^ ions in the internal solution for equimolar Cs^+^ ions, and the pH value in the solution was titrated to 7.2 by adding CsOH. To avoid the contamination of Cl^−^ currents, Cl^−^ ions inside the pipette solution were replaced with aspartate. All solutions used for this study were generally prepared in deionized water from a Milli-Q^®^ water purification system (Merck Millipore, Taipei, Taiwan). The pipette solution and culture media were always filtered with an Acrodisc^®^ syringe filter that contains a 0.2-μm Supor^®^ nylon membrane (#4612; Pall Corp.; Genechain Biotechnology, Kaohsiung, Taiwan).

### 4.2. Cell Preparations

The GH_3_ pituitary cell line was supplied by the Bioresources Collection and Research Center (BCRC-60015; Hsinchu, Taiwan), whereas the embryonic mouse hippocampal cell line (mHippoE-14, CLU198) was by Codarlane CELLutions Biosystems, Inc. (Burlington, ON, Canada). The GH_3_ cell line was originally derived from the American Type Culture Collection (ATCC^®^ [CCL-82.1TM]; Manassas, VA, USA). The GH_3_ cell line was maintained by growing cells in 50-mL plastic culture flasks in 5 mL of Ham’s F-12 medium, supplemented with 2.5% fetal calf serum (*v*/*v*), 15% horse serum (*v*/*v*), and 2 mM L-glutamine, while mHippoE-14 neurons were in Dulbecco’s modified Eagle’s medium, supplemented with 10% fetal bovine serum (*v*/*v*) and 2 mM L-glutamine. Flasks were kept at 37 °C in a humidified environment of 5% CO_2_/95% air. Growth medium was replaced twice a week, and cells were split into subcultures once a week. Electrophysiological measurements were made 6–10 days after the plating of the subcultures.

### 4.3. Electrophysiological Measurements

On the day of the experiments, we dispersed GH_3_ cells or mHippoE-14 neurons with 1% trypsin/EDTA solution, and a few drops of cell suspension was placed in a home-made recording chamber positioned on the stage of an inverted Olympus fluorescence microscope (CKX-41; Yuan Yu, Taipei, Taiwan). The microscope was set on an antI–Vibration air table contained within a Faraday’s cage, and it was also coupled to a digital video system (DCR-TR30; Sony, Tokyo, Japan), with a magnification of up to 1500×, to continuously monitor changes in both cell size and the electrode’s position. Cells were kept immersed at room temperature (20–25 °C) in normal Tyrode’s solution containing 1.8 mM CaCl_2_, and the solution’s composition is elaborated above. After the cells were allowed to adhere to the chamber’s bottom for several minutes, the recordings were carried out. The patch-clamp procedure in whole-cell mode (i.e., when membrane patch was broken by suction) was performed by using either an RK-400 (Biologic, Echirolles, France) or an Axopatch-200 amplifier (Molecular Devices; Bestogen Biotech, New Taipei City, Taiwan) [67]. When filled with internal solution, the pipettes used for recording had tip resistances ranging from 3 to 5 MΩ, and they were fabricated from Kimax-51 borosillicate capillaries (#34500 [1.5–1.8 mm in outer diameter]; Dogger, Tainan, Taiwan) by using either a PP-830 vertical puller (Narishige, Tokyo, Japan) or a P-97 programmable horizontal puller (Sutter, Novato, CA, USA) and the electrodes’ tips were fire-polished with an MF-83 microforge (Narishige). The potentials were corrected for the liquid-liquid junction potential, which develops when the composition of the pipette solution is different from that in the bath. Tested compounds were either applied through perfusion or added to the bath in the attempts to achieve the final concentration indicated.

### 4.4. Data Recordings

The signals were monitored on an HM-507 oscilloscope (Hameg, East Meadow, NY, USA) and digitally stored online in an ASUS ExpertBook laptop computer (P2451F; ASUS, Tainan, Taiwan) at 10 kHz, interfaced with a Digidata 1440A converter (Molecular Devices; Bestogen Biotech, New Taipei City, Taiwan). The latter device was used for efficient analog-to-digital/digital-to-analog (AD/DA) conversion. During the measurements, the process in data acquisition, equipped with this device, was controlled by pCLAMP 10.6 software (Molecular Devices) run under Windows 7 (Redmond, WA, USA), and the signals were simultaneously displayed on an LCD monitor through a USB type-C connection. Current signals that we achieved were low-pass-filtered at 2 kHz with an FL-4 four-pole Bessel filter (Dagan, Minneapolis, MN, USA) to minimize possible electrical interference. After the recorded data were digitally acquired, we off-line collated them using various analytical tools that included the LabChart 7.0 program (ADInstruments; Gerin, Tainan, Taiwan), OriginPro^®^ 2021 (OriginLab; Scientific Formosa, Kaohsiung, Taiwan), and different custom-made macros built in Excel^®^ 2021 under Office 365 (Redmond, WA, USA). pCLAMP-generated voltage-clamp profiles, in which either rectangular or ramp waveforms were created, were employed to examine the current-voltage (*I–V*) relationship, the steady-state inactivation curve, the recovery time course of current inactivation, or the voltage-dependent hysteresis (V_hys_) of ionic currents (e.g., *I*_K(A)_).

### 4.5. Whole-Cell Current Analyses

To determine the concentration-dependent inhibition of LCS on the peak or sustained component of macroscopic *I*_K(A)_, GH_3_ cells were immersed in Ca^2+^-free, Tyrode’s solution in which 1 μM TTX and 0.5 mM CdCl_2_ were added. The examined cell was held at −80 mV and 1 s depolarizing step from −80 until a clamping potential of −30 mV was delivered to it. Current amplitudes (at the beginning- and end-pulse of command voltage) evoked in response to depolarizing pulses to −30 mV were acquired in the control period (i.e., LCS was not present) and during the exposure to varying LCS concentrations (3 μM–1 mM). The concentration required to suppress 50% (i.e., IC_50_) of peak or sustained *I*_K(A)_ was calculated with the goodness of fit by the use of a modified form of the Hill equation. That is,

$$Relative\;amplitude=\frac{{\left[LCS\right]}^{-n_{H}}\times \left(1-a\right)}{IC_{50}^{-n_{H}}+{\left[LCS\right]}^{-n_{H}}}+a$$

where [*LCS*] represents the different concentrations of LCS used; n_H_ and *IC*_50_ are, respectively, the Hill coefficient inherent to the concentration–response response and the concentration at which 50% inhibition of peak or sustained *I*_K(A)_ is observed. At this point, maximal inhibition (1−*a*) of peak or sustained *I*_K(A)_ is also evaluated.

The time-dependent rate constants of the blocking (forward, on, *k*_+1_*) or unblocking (backward, off, *k*_−1_) reaction, taken with or without the application of different LCS concentrations, were evaluated from the slow component of the inactivation time constant (*τ_inact(S)_*) of *I*_K(A)_ evoked by depolarizing command voltage from −80 to −30 mV with a duration of 1 s. The *τ_inact(S)_* values obtained in the presence of different LCS concentrations were approximated by fitting a two-exponential function (i.e., fast and slow components) to the decaying trajectory of each current trace. Since a Hill coefficient of about 1 was noticed according to the concentration-dependent relationship, the forward or backward rate constant was then extended, as determined using the following equation (i.e., simple linear regression model):

1τinactS=k−1+k+1*·LCS

in which [*LCS*] is the known concentration of LCS, and *k*_+1_^*^ or *k*_−1_ is respectively achieved from the slope (i.e., Δ(1/*τ_inact(S)_*)/Δ[*LCS*]) or the vertical y-axis intercept at [*LCS*] = 0 of the interpolated regression line (i.e., the point at which the line crosses the y-axis), where the relation of the reciprocal time constant of *I*_K(A)_ inactivation (i.e., 1/*τ_inact(S)_*) versus the LCS concentration was constructed.

The relationship of the conditioning potential versus the *I*_K(A)_ amplitude acquired with or without the LCS addition (i.e., quasi-steady-state inactivation curve of *I*_K(A)_) was approximated by a modified Boltzmann function (or the Fermi-Dirac distribution) of the following form:

IImax=11+exp(V−V12)qFRT

where *I_max_* is the maximal amplitude of *I*_K(A)_, *V*_1/2_ the voltage (in mV) at which half-maximal inactivation of the current is achieved, *q* the apparent gating charge in *e* (i.e., elementary charge), and *F*, *R,* and *T* the Faraday’s constant, the universal gas constant, and the absolute temperature, respectively.

### 4.6. Curve-Fitting Procedures and Statistical Analyses

Linear (e.g., relation of 1/*τ_inact(S)_* versus the LCS concentration) or nonlinear (e.g., Hill or Boltzmann equation and double exponential) curve fitting to the experimental data sets presented herein was performed with the least-squares fit by using either the Solver add-in bundled with Excel^®^ 2021 (Microsoft) or OriginPro^®^ 2021 (OriginLab). The values are provided as means ± standard error of mean (SEM) with sample sizes (n), which denotes the cell number carefully collected. Student’s *t*-test (paired or unpaired samples) or analysis of variance (ANOVA-1 or ANOVA-2), followed by post-hoc Fisher’s least-significance difference test for multiple-range comparisons, was implemented for the statistical evaluation. Probability with *p* < 0.05 was considered statistically significant.

## Data Availability

The datasets used in this study are available on reasonable request.

## Supplementary Materials

The following are available online at www.mdpi.com/article/10.3390/ijms23031171/s1.

## Abbreviations

ANOVA	analysis of variance
4-AP	4-aminopyridine
4-AP-3-MeOH	4-aminopyridine-3-methanol
Cap	capsaicin
*I*_K(A)_	A-type K^+^ current
*I–V*	current voltage
IC_50_	the concentration required for 50% inhibition
K_A_	A-type K^+^ channel
*K*_D_	dissociation constant
K_V_ channel	voltage-gated K^+^ channel
LCS	lacosamide (Vimpat^®^)
Na_V_ channel	voltage-gated Na^+^ channel
SEM	standard error of mean
TEA	tetraethylammonium chloride
τ*_inact(S)_*	time constant in the slow component of current inactivation
TTX	tetrodotoxin
V_hys_	voltage-dependent hysteresis
