# Supplementary material for: Evidence for Inhibitory Perturbations on the Amplitude, Gating, and Hysteresis of A-Type Potassium Current, Produced by Lacosamide, a Functionalized Amino Acid with Anticonvulsant Properties
Source: Int J Mol Sci. 2022 Jan 21;23(3):1171. doi: 10.3390/ijms23031171 (PMC8835568; doi:10.3390/ijms23031171)
Supplement: Supplementary file 1 [file ijms-23-01171-s001.zip › ijms-1522991-supplementary.pdf]

Supplementary Information

The time course of LCS-induced reduction in the peak and sustained of $I_{K(A)}$ identified in GH_3 cells

In these experiments, the examined cell was voltage-clamped at -80 mV and the depolarizing pulse from -80 to -30 mV with a duration of 1 sec. The peak and sustained components of $I_{K(A)}$ in response to membrane depolarization were measured during cell exposure to 100 μM LCS (Supplementary Figure 1).

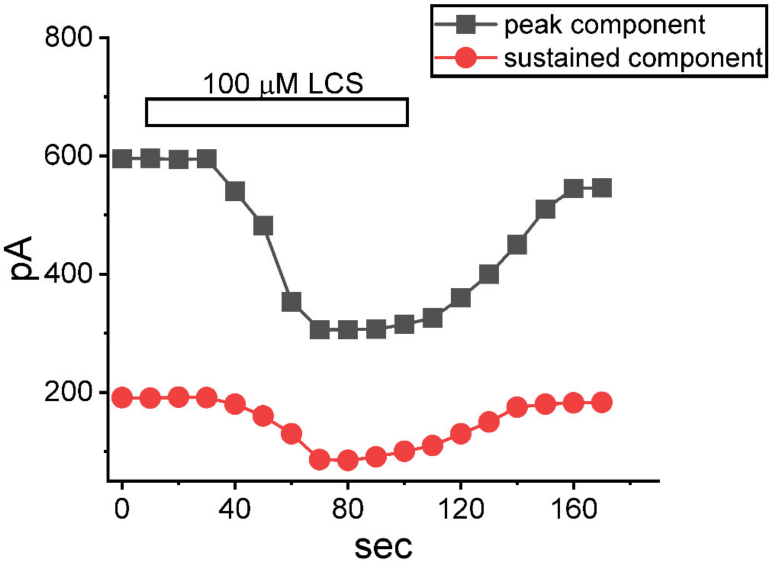

Figure S1. Time course showing inhibitory effect of LCS on peak (filled black symbols) and sustained (filled red symbols) components of $I_{K(A)}$ evoked by 1-sec membrane depolarization. Cells were bathed in Ca^{2+}-free, Tyrode's solution, and the electrode filled with K^+-containing solution. The horizontal bar shown above indicates the presence of 100 μM LCS. Current amplitude was taken at the start or end of each depolarizing pulse.
